# Maternal socioeconomic status and infant mortality with low birth weight as a mediator: an inter-country comparison between Scotland and Denmark using administrative data

**DOI:** 10.23889/ijpds.v1i1.368

**Published:** 2017-04-19

**Authors:** Yongfu Yu, Maximiliane Verfuerden, Pia Hardelid, Ania Zylbersztejn, Zeyan Liew, Jørn Olsen, Ruth Gilbert, Jiong Li, Bo Fu

**Affiliations:** 1 Section for Epidemiology, Department of Public Health, Aarhus University; 2 University College London, Institute of Child Health; 3 Administrative Data Research Centre for England; 4 Department of Epidemiology, Fielding School of Public Health, University of California; 5 Department of Clinical Epidemiology, Aarhus University Hospital

## Objectives

Higher rates of infant mortality in the UK than in the Nordic countries are partly explained by wider socio-economic disparities in the UK. We examined the extent to which low birth weight mediates the association between socioeconomic status (SES) and infant mortality using causal mediation analysis. We used cohorts of live births identified in administrative hospital data for the whole of Scotland and Denmark to explore the contribution of prenatal factors, represented by low birth weight, to differences in infant mortality between the two countries.

## Approach

We included live-born children born in Denmark (n=1,432,205) and Scotland (n=1,427,163) from 1981-2004. Follow up was to 12 months of age. Information on deaths in first year of life was obtained through linkage with cause of death registers. We determined the effect of socioeconomic status on all cause infant mortality by comparing the highest and lowest quintiles of area-based deprivation (based on Carstairs score in Scotland) or level of maternal education in Denmark. Causal mediation analysis was used for survival outcomes with adjustment for maternal age at birth, sex, birth year of the child, and records indicating congenital malformation.

## Results

During the follow-up, there were 8,158(0.57%) deaths in Denmark and 8,271(0.58%) deaths in Scotland. Comparing with the very high SES group, the overall hazard ratios of death for each SES quintile (starting with the lowest) compared with the highest SES quintile were 1.58(95% Confidence interval: 1.47-1.71), 1.40(1.32-1.49),1.25(1.20-1.30), 1.11(1.09-1.14) in Denmark, and 1.50(1.36-1.65),1.35(1.25-1.45),1.22(1.16-1.28),1.10(1.08-1.13) in Scotland. The proportions of excess infant deaths mediated through low birth weight (starting with the lowest) compared with the highest SES quintile were 54.7%, 52.1%, 49.5%, 46.9% in Denmark, and 26.0%, 23.9%, 22.0%, 20.1% in Scotland.

## Conclusion

Our result suggests that SES has similar effects on infant mortality in Denmark and Scotland but more of the effect of SES on infant mortality is mediated through low birth weight in Denmark. Public health preventive strategies for infant mortality in both countries need to address prenatal risk factors for low birth weight. The substantial direct effects of SES on infant mortality seen in Scotland, which were not mediated through low birth weight, may be explained by other birth characteristics or could reflect persisting SES disparities in the care of infants after birth. 

